# Integrated Metabolic and Micronutrient Dynamics Across the First Postoperative Year: Interval-Based Profiling in 340 Brazilian Women Undergoing Sleeve Gastrectomy

**DOI:** 10.1007/s11695-026-08850-8

**Published:** 2026-07-22

**Authors:** Alana Bonfim de Oliveira, Gabrielly Eduarda Piovezan Pereira, Eduarda Martinowski Henriques Guia, Otávio Sales, Thamira Fonteles de Moura, Wilson Paulo dos Santos, Mônica Maciel, Lucélia Donatti, Luiz Claudio Fernandes, Katya Naliwaiko, Mariana Inocêncio Manzano Watanabe, Matheus Felipe Zazula

**Affiliations:** 1 Grupo de Pesquisa em Gestação e Doenças Metabólicas, Centro Universitário Cesumar, Curitiba, Brazil; 2 Departamento de Biologia Celular, Universidade Federal do Paraná, Curitiba, Brazil; 3 Clinica WPS de Cirurgia Bariátrica, Curitiba, Brazil; 4 Departamento de Biologia Celular, Universidade Federal do Paraná, Curitiba, Brazil; 5 Departamento de Fisiologia, Universidade Federal do Paraná, Curitiba, Brazil

**Keywords:** Sleeve Gastrectomy, Micronutrient Deficiency, Interval-based Profiling, Postoperative Nutrition, Metabolic Profiling

## Abstract

**Aims:**

This study aimed to characterize the temporal dissociation between metabolic recovery and nutritional status during the first postoperative year following sleeve gastrectomy in women.

**Materials and methods:**

This retrospective cohort study evaluated 340 women undergoing sleeve gastrectomy using laboratory data from routine clinical follow-up. Biochemical markers were assessed preoperatively and at approximately 40, 120, 180, and 365 days postoperatively. A comprehensive multisystem panel was used to track longitudinal changes in nutritional, haematologic, metabolic, hepatic, inflammatory, renal, and endocrine parameters using an interval-based analytical approach.

**Results:**

Distinct postoperative trajectories were identified between metabolic recovery and nutritional status. Metabolic and inflammatory parameters improved rapidly and remained stable throughout follow-up, including reductions in fasting glucose, triglycerides, liver enzymes, uric acid, and C-reactive protein, alongside sustained increases in HDL cholesterol. In contrast, several nutritional biomarkers showed progressive deterioration. Folate exhibited the earliest and most pronounced decline, decreasing by more than two-thirds within 40 days and remaining persistently reduced throughout follow-up. Vitamin B12 declined progressively, reaching concentrations compatible with early biochemical insufficiency. Ferritin showed continuous depletion across all intervals, whereas serum iron increased, indicating dissociation between circulating iron and iron stores. Zinc concentrations declined sharply and remained persistently reduced, while albumin and globulins exhibited modest but consistent decreases. Multivariate analyses confirmed coordinated metabolic–hepatic remodelling occurring in parallel with a weaker and progressively deteriorating nutritional trajectory.

**Conclusions:**

Metabolic recovery and nutritional adequacy represent partially dissociated physiological processes following sleeve gastrectomy. Micronutrient deterioration begins early and progresses before overt clinical manifestations, underscoring the need for frequent biochemical monitoring and individualized supplementation strategies during postoperative follow-up.

## Introduction

Obesity is a globally prevalent and biologically complex condition characterized by dysregulated adipose tissue biology, chronic low-grade systemic inflammation, and multisystem metabolic impairment [26, 60]. Its development involves interactions among genetic, neuroendocrine, environmental, and behavioural factors, resulting in progressive metabolic deterioration and increased long-term morbidity [17, 26, 63]. Although lifestyle interventions remain the cornerstone of obesity management, they often fail to achieve sustained weight loss in individuals with severe obesity, making bariatric surgery an important therapeutic option for long-term metabolic improvement [43].

Sleeve gastrectomy has become the most performed bariatric procedure worldwide and is increasingly recognized as a metabolic intervention rather than a purely restrictive operation [16, 45]. By removing approximately 70 to 80% of the gastric reservoir, sleeve gastrectomy alters gastric compliance, accelerates nutrient transit, and modifies vagal and enteroendocrine signalling. These anatomical and functional changes result in marked reductions in ghrelin secretion and enhanced activity of incretin hormones such as glucagon like peptide 1 and peptide YY, contributing to sustained weight loss and improvements in glycaemic control, lipid metabolism, hepatic steatosis, and systemic inflammatory tone [2, 8, 38, 51]. Sleeve gastrectomy is primarily indicated for individuals with severe obesity and obesity-associated comorbidities when conventional therapeutic approaches fail to achieve sustained weight reduction.

Despite these metabolic benefits, sleeve gastrectomy substantially alters the biochemical environment required for optimal digestion, absorption, and utilization of micronutrients. Reduced gastric acidity impairs the dissociation of protein bound vitamins and minerals, diminished intrinsic factor secretion compromises vitamin B12 absorption, and postoperative dietary restriction limits the intake of nutrient dense foods. In parallel, rapid weight loss, postoperative inflammatory modulation, and interindividual variability in supplementation adherence further contribute to nutritional vulnerability, even in the absence of intestinal bypass [9, 25, 35, 59].

Micronutrients essential for erythropoiesis, one-carbon metabolism, oxidative balance, immune competence, and musculoskeletal integrity, including iron, ferritin, folate, vitamin B12, vitamin D, calcium, and zinc, are particularly susceptible to postoperative disruption. Importantly, subclinical deficiencies in these elements may develop well before overt clinical manifestations such as anaemia, neuropathy, osteometabolic disease, or impaired immune function become apparent. Women represent a population of relevance in this context due to sex specific differences in iron metabolism, baseline micronutrient reserves, and reproductive physiology, which may amplify postoperative nutritional risk [11, 22, 34, 48].

Although nutritional alterations following bariatric surgery are well recognized, existing evidence remains constrained by important methodological limitations. Many available studies rely on small sample sizes, cross sectional designs, or restricted biochemical panels, which limit the ability to characterize the temporal sequencing, magnitude, and multisystem integration of postoperative nutritional change [22, 55]. Moreover, longitudinal investigations capable of capturing coordinated physiological remodelling across metabolic and nutritional domains, particularly during the early postoperative period, remain scarce [11]. This limitation is especially pronounced in cohorts of women undergoing sleeve gastrectomy, a population that is frequently underrepresented in high resolution nutritional studies despite its clinical relevance [12, 48]. Consequently, the relationship between metabolic recovery and nutritional adequacy during the first postoperative year remains insufficiently characterized.

Addressing these gaps requires an approach capable of integrating haematologic, metabolic, endocrine, inflammatory, and nutritional biomarkers across multiple postoperative intervals. The present study applies such a strategy by providing a comprehensive, interval-based characterization of biochemical trajectories during the first year following sleeve gastrectomy in women. By examining the temporal dissociation between metabolic improvement and nutritional status, this work aims to clarify the onset, progression, and persistence of micronutrient deterioration in a real-world clinical context. We hypothesised that sleeve gastrectomy, despite promoting substantial metabolic recovery, is associated with early, coordinated, and progressive depletion of key micronutrient and protein reserves that remain partially dissociated from metabolic improvement and precede overt clinical deficiency.

## Materials and methods

### Study Design

This study is an observational retrospective cohort analysis based on the systematic review of electronic medical records from women who underwent sleeve gastrectomy between 2020 and 2025. The primary objective was to characterize interval-based biochemical trajectories of metabolic and micronutrient markers during the first postoperative year using data derived from routine clinical follow-up. The clinical data were kept in accordance with Brazilian law [33].

Although the study spans a 12-month postoperative period, it was not designed as a fully repeated measures longitudinal analysis at the individual level. Due to heterogeneity in follow-up schedules inherent to real-world clinical practice, not all participants contributed laboratory data at all postoperative intervals. Therefore, analyses were conducted using predefined postoperative intervals as independent analytical groups rather than subject specific repeated measures models. This approach prioritizes clinical representativeness and maximizes sample size within each interval while acknowledging variability in individual follow-up completeness.

Patients contributing laboratory data to later postoperative intervals may represent a clinically distinct subgroup, potentially enriched for individuals with greater adherence to follow-up, persistent clinical monitoring, postoperative complications, or suspected nutritional deficiencies. Therefore, follow-up related selection bias inherent to retrospective real-world cohorts should be considered when interpreting long-term biochemical trajectories.

Postoperative intervals were defined a priori based on clinically meaningful phases of postoperative adaptation and included assessments at approximately 40-, 120-, 180-, and 365-days following surgery. A preoperative baseline was included as the biological reference condition for all analyses. All laboratory data were obtained as part of standard postoperative monitoring and reflect routine care rather than protocol driven sampling.

### Ethical Approval and Data Protection

Patient confidentiality was ensured through full anonymization of personal identifiers prior to data extraction and analysis. Data access was restricted to authorized researchers, and all analyses were performed using de-identified datasets derived exclusively from routine clinical records. *Ethical approval details are provided on the title page.*

### Participants

Eligible participants were women aged between 18 and 50 years who underwent sleeve gastrectomy and had at least one complete laboratory panel available within the predefined postoperative intervals. Inclusion was restricted to individuals with verified surgical dates and clearly documented postoperative timelines to ensure accurate interval classification.

Exclusion criteria included prior bariatric or gastrointestinal surgery, chronic liver disease, advanced renal insufficiency, and uncontrolled endocrine disorders, including uncontrolled thyroid diseases, active Cushing’s syndrome, acromegaly, and other endocrinopathies capable of directly influencing the biochemical markers evaluated in the present study. Obesity-associated type 2 diabetes under pharmacological treatment and regular clinical follow-up was not considered an exclusion criterion. Patients with incomplete, inconsistent, or implausible laboratory records were also excluded following data quality assessment.

The requirement of at least one complete laboratory panel was adopted to maximize sample size while preserving internal consistency of biochemical analyses within each interval. This strategy reflects the inherent heterogeneity of follow-up in real-world bariatric care and prioritizes representativeness over complete longitudinal sampling at the individual level [40].

This study included only biological females, defined by sex assigned at birth, which limits the generalizability of the findings to other populations. This retrospective cohort included women with obesity and medically controlled diabetes undergoing sleeve gastrectomy, using exclusively information available from clinical records and laboratory examinations obtained during routine postoperative care. Severe associated comorbidities, when identified in the clinical records, were not included in the analyses. Detailed information regarding mobility status, behavioural risk factors, and genetic causes of obesity was not systematically available in the retrospective database and therefore could not be consistently evaluated within the scope of the present study. The preoperative baseline group was used as the biological reference condition for all analyses. Consequently, the present study was designed to evaluate interval-based postoperative trajectories rather than to estimate the prevalence of micronutrient deficiencies within the source population.

### Data Collection and Clinical Records

Data were extracted from electronic clinical records in collaboration with a specialized private bariatric surgery clinic located in Brazil, reflecting routine postoperative follow-up performed within a real-world clinical setting. Demographic information, surgical details, and postoperative follow-up dates were verified manually to ensure accurate temporal classification of participants across the predefined postoperative intervals.

Laboratory data were obtained exclusively from tests requested as part of routine postoperative clinical care. All analyses were performed in accredited clinical laboratories, and only results generated using validated analytical methods were included. After extraction, laboratory values were harmonized into a standardized database and subjected to quality control procedures, including consistency checks, identification of implausible values, and assessment of missing data patterns.

Data handling and preprocessing were conducted using de-identified datasets. No additional laboratory testing or protocol driven sampling was performed for the purposes of this study, ensuring that the findings reflect real-world postoperative monitoring practices.

### Preoperative Baseline and Postoperative Assessment Periods

Biochemical markers were evaluated across five predefined intervals to characterize temporal patterns following sleeve gastrectomy using an interval-based analytical framework. These intervals comprised a preoperative baseline and postoperative assessments conducted at 40, 120, 180, and 365 days after surgery. The selection of these timepoints was guided by their clinical relevance and by their capacity to capture distinct phases of postoperative physiological adaptation, while allowing standardized comparison across groups in the context of heterogeneous real-world follow-up.

The preoperative baseline was defined as the reference condition for all analyses and reflects the metabolic, inflammatory, and nutritional status of participants prior to surgical intervention. Inclusion of a baseline assessment was essential to account for pre-existing biochemical variability commonly observed in individuals with obesity and to provide a consistent biological reference against which postoperative changes could be evaluated.

The postoperative assessment at 40 days was selected to capture early physiological changes occurring during the initial postoperative phase, which is characterized by rapid weight loss, substantial dietary restriction, and marked alterations in gastrointestinal function. This interval allows identification of early postoperative biochemical shifts across metabolic and nutritional domains without presupposing their direction or persistence.

Assessments conducted at 120 and 180 days after surgery were included to evaluate biochemical profiles during intermediate postoperative phases, encompassing early adaptation and subsequent consolidation of physiological changes. These intervals enable examination of whether biochemical alterations observed shortly after surgery persist, attenuate, or stabilize over time within an interval-based analytical structure.

The 365-day postoperative assessment was defined to characterize biochemical profiles during the later postoperative period, when weight loss typically slows and longer-term physiological adaptations become established. Inclusion of this interval allows evaluation of sustained postoperative biochemical patterns within the first year following sleeve gastrectomy.

Together, integration of these predefined intervals enables standardized temporal mapping of postoperative biochemical profiles across distinct phases of recovery using an interval-based approach, while maintaining methodological consistency in the presence of variable individual follow-up schedules.

### Laboratory Measures

A comprehensive biochemical panel was selected to enable multisystem characterization of postoperative physiology following sleeve gastrectomy, with particular emphasis on nutritional, haematologic, metabolic, hepatic, renal, inflammatory, and endocrine domains. Because this was a retrospective study based on routine clinical records, detailed information regarding analytical platforms, reagents, calibration procedures, and assay-specific methodologies was not consistently available. However, all examinations were performed in accredited clinical laboratories operating under Brazilian regulatory standards established by ANVISA and the Ministério da Saúde, using validated methodologies routinely employed in clinical practice. Analyses were conducted in accredited clinical laboratories certified by the Ministério da Saúde and compliant with ANVISA [47], ensuring methodological standardization, analytical validity, and comparability across patients and postoperative intervals.

Haematologic assessment comprised complete blood count parameters, including hemoglobin concentration, haematocrit, red blood cell count, erythrocyte indices, leukocyte counts, and platelet counts. These variables were included to capture alterations in erythropoiesis and overall haematologic status potentially associated with postoperative nutritional changes. Iron status was evaluated using serum iron and ferritin concentrations, allowing parallel assessment of circulating iron availability and stored iron reserves.

Micronutrient status was assessed using measurements of folate, vitamin B12, vitamin D, and zinc, when available. These micronutrients were selected due to their established relevance to one-carbon metabolism, erythropoiesis, immune function, oxidative balance, and neuromuscular integrity, as well as their known susceptibility to postoperative alteration following bariatric surgery. Protein nutritional status was evaluated using serum albumin, total protein, and globulin fractions, enabling assessment of systemic protein reserve and proportional shifts among serum protein components.

Metabolic status was characterized using fasting glucose and glycated hemoglobin as markers of glycaemic regulation, along with total cholesterol, high density lipoprotein cholesterol, and triglycerides to evaluate lipid metabolism. Hepatic function was assessed using serum activities of aspartate aminotransferase, alanine aminotransferase, and gamma glutamyl transferase, which together provide complementary information on hepatocellular integrity and cholestatic activity. Systemic inflammatory status was evaluated using C reactive protein.

Renal and endocrine function were assessed using serum creatinine, thyroid stimulating hormone, and free thyroxine concentrations, enabling evaluation of renal filtration markers and thyroid axis activity across postoperative intervals. Mineral balance was assessed using total calcium measurements, allowing integration with protein markers in subsequent analyses.

Together, this panel was selected to support integrated evaluation of postoperative biochemical adaptation across multiple physiological systems using an interval-based analytical approach, without reliance on protocol driven sampling or experimental manipulation. This strategy enables characterization of real-world postoperative biochemical trajectories following sleeve gastrectomy within the first postoperative year.

### Statistical Analysis

All statistical analyses were performed using R software version 4.5.1 [46]. Data inspection was conducted prior to inferential testing to evaluate distributional properties and analytical assumptions. Normality was assessed using the Shapiro Wilk test [50] in combination with visual inspection of density plots and quantile plots. Homogeneity of variances was evaluated using Levene’s test [58] alongside examination of residual dispersion patterns. These procedures were applied to guide the selection of appropriate univariate analytical methods without assuming distributional symmetry across biochemical markers.

For variables that met parametric assumptions, comparisons across postoperative intervals were performed using one-way ANOVA. When heteroscedasticity was detected, heteroscedasticity consistent covariance estimators were applied. Post hoc comparisons were conducted using Tukey’s adjustment. Variables violating normality assumptions were analysed using the Kruskal Wallis test, with pairwise comparisons corrected using the Benjamini Hochberg false discovery rate procedure [6]. Descriptive data are presented as means with standard deviations, medians with interquartile ranges, or proportions with corresponding confidence intervals, as appropriate. All statistical tests were two sided, adopting a significance threshold of 0.05, with emphasis placed on effect size patterns and confidence intervals rather than isolated p values.

To evaluate postoperative changes as integrated physiological systems rather than isolated biochemical markers, a multivariate analytical framework was adopted. Given the heterogeneous distributions, collinearity structure, and unequal variance characteristics of clinical biochemical data, parametric multivariate models such as MANOVA were considered inappropriate. Instead, permutational multivariate analysis of variance was employed using the adonis2 function from the vegan package. Prior to multivariate analysis, biochemical variables were standardized using z scores and grouped into predefined physiological panels reflecting coherent biological domains, including haematologic markers, iron status, protein markers, vitamins and minerals, metabolic markers, hepatic function and inflammation, and renal and endocrine function. For each panel, Euclidean distance matrices were computed. Multivariate models were specified as distance matrix as a function of postoperative group, using marginal sums of squares and 10,000 permutations. Because PERMANOVA may be influenced both by differences in centroid location and by heterogeneity of multivariate dispersion among groups, interpretation of significant multivariate effects considered both structural separation and potential dispersion-related contributions across postoperative intervals [18, 20, 42, 57].

The preoperative baseline group was included as the biological reference condition in all multivariate analyses. Inferential interpretation focused on the magnitude and persistence of divergence from baseline across postoperative intervals rather than symmetric group to group comparisons. When global permutational tests indicated significant group effects, pairwise post hoc permutational analyses were conducted by subsetting distance matrices for each pair of groups and reapplying the same analytical framework. Resulting p values were adjusted using Bonferroni correction to control for family wise error.

To visualize multivariate structure and temporal patterns, principal coordinates analysis was applied to each physiological panel using the corresponding distance matrices. Ordination scores from the first two principal coordinates were used to generate two-dimensional representations of group separation. Convex hulls were constructed to illustrate within group dispersion and between group separation in multivariate space. These ordination plots were used descriptively to support interpretation of permutational results rather than as inferential tools.

Exploratory principal component analysis was additionally applied to the set of biochemical variables showing significant variation across postoperative intervals. Sampling adequacy was assessed using the Kaiser Meyer Olkin statistic and Bartlett’s test of sphericity. Principal component analysis was performed on standardized variables using a correlation-based approach, and component interpretation was guided by eigenvalues, explained variance, and variable loadings. Finally, multivariate relationships among physiological panels were explored using scatterplot matrices of ordination scores, enabling assessment of coordinated or dissociated system level responses across postoperative intervals. Correlation analyses among panel scores were conducted using Pearson correlation coefficients. These exploratory analyses were intended to complement univariate and permutational findings by providing an integrated view of postoperative physiological remodelling [29, 30, 57].

## Results

### Cohort Characteristics

**Fig. 1:**
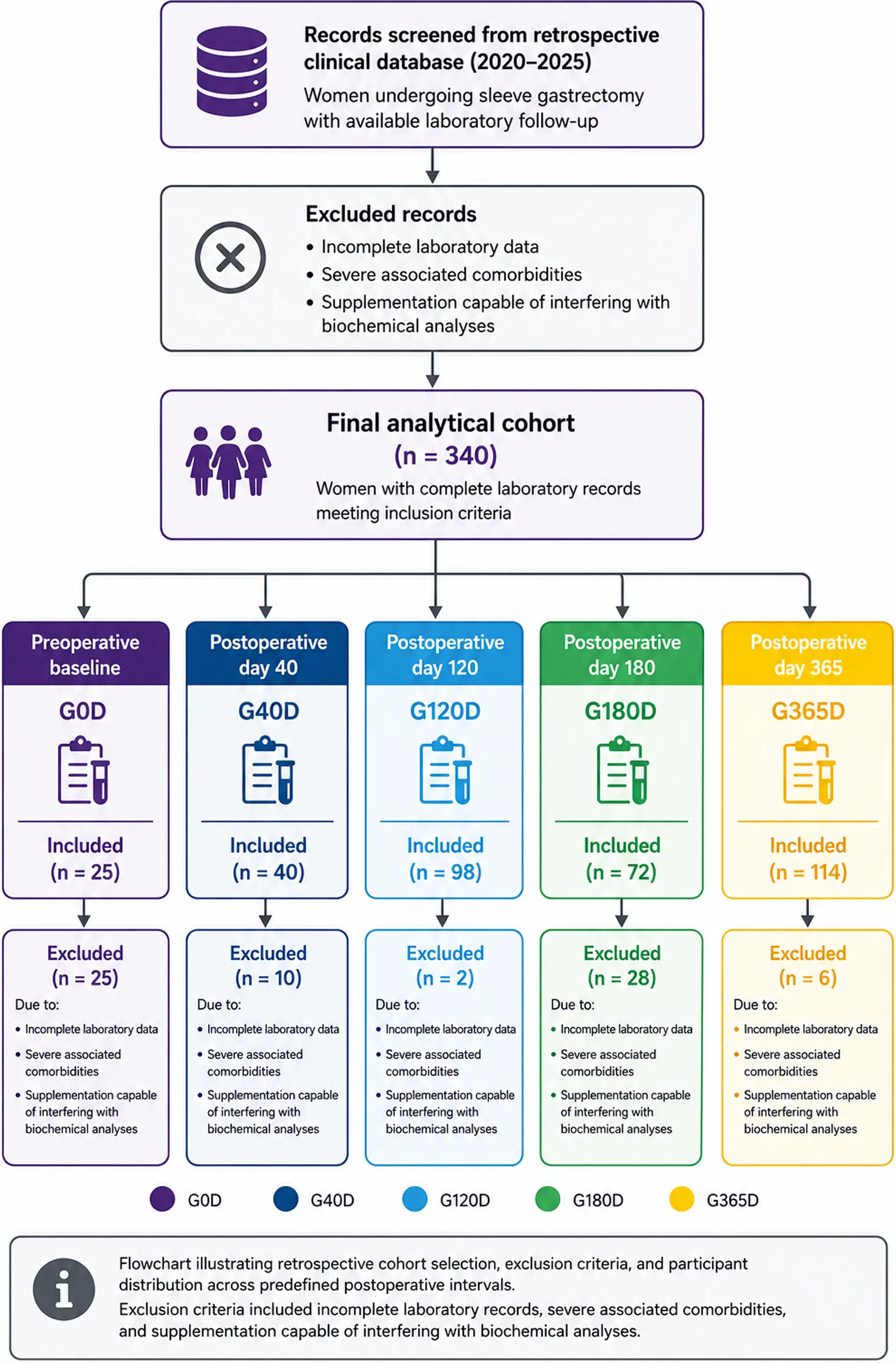
Flowchart illustrating retrospective cohort selection, exclusion criteria, and participant distribution across predefined postoperative intervals. Eligible records included women undergoing sleeve gastrectomy with available laboratory follow-up data obtained during routine postoperative care. Exclusion criteria comprised incomplete or inconsistent laboratory records, severe associated comorbidities, and active targeted supplementation capable of directly interfering with the biochemical analyses corresponding to the evaluated interval. Final analytical groups were distributed according to predefined postoperative intervals: preoperative baseline (G0D), postoperative day 40 (G40D), postoperative day 120 (G120D), postoperative day 180 (G180D), and postoperative day 365 (G365D)

A total of 340 women who had undergone vertical sleeve gastrectomy and possessed complete laboratory records were included in the analysis. Participants were distributed across predefined postoperative intervals as follows: preoperative baseline (G0D, *n* = 25), 40 days (G40D, *n* = 40), 120 days (G120D, *n* = 98), 180 days (G180D, *n* = 72), and 365 days (G365D, *n* = 114). A detailed flowchart describing retrospective cohort selection, exclusion criteria, and participant distribution across predefined postoperative intervals is presented in Fig. 1. The flowchart also details exclusions related to active targeted supplementation, incomplete laboratory records, and severe associated comorbidities.

**Fig. 2:**
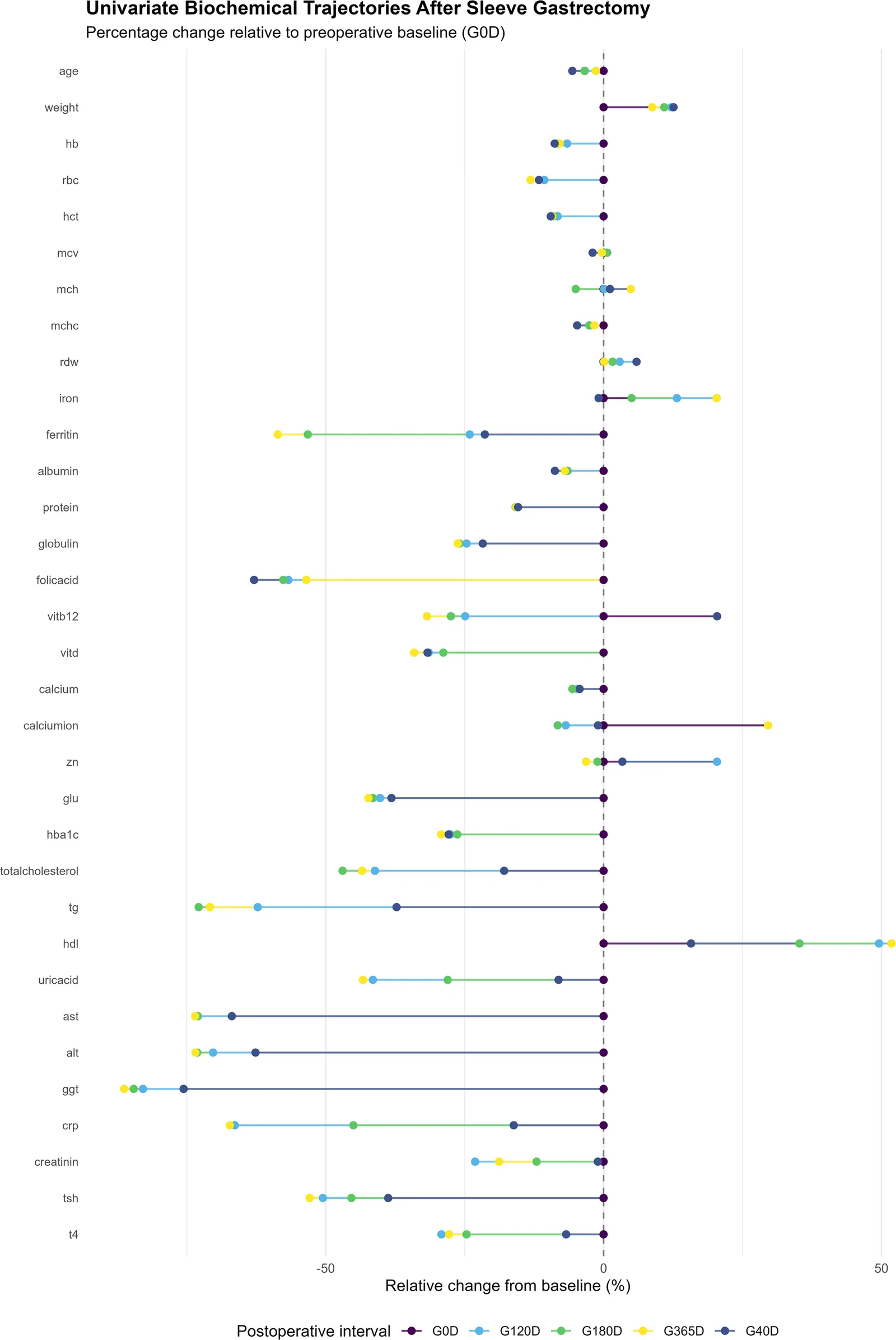
Univariate biochemical trajectories relative to the preoperative baseline following sleeve gastrectomy. Changes in individual biochemical, haematological, nutritional, metabolic, hepatic, renal, and endocrine markers are expressed as percentage variation relative to the preoperative baseline (G0D). Colored points represent group means at each postoperative interval: 40 days (navy), 120 days (light blue), 180 days (green), and 365 days (yellow). The preoperative baseline (G0D) is shown as the reference value (0%, purple). Horizontal segments connect baseline values to postoperative means, illustrating the magnitude and direction of change over time. The vertical dashed line denotes no change relative to baseline. Positive values indicate increases, whereas negative values indicate reductions relative to the preoperative state. Variables are ordered according to their physiological grouping and appearance in the Results section, allowing integrated visualization of coordinated metabolic improvement alongside progressive alterations in micronutrient, protein, haematological, hepatic, renal, and endocrine parameters across the first postoperative year. HB, haemoglobin; RBC, red blood cell count; HCT, haematocrit; MCV, mean corpuscular volume; MCH, mean corpuscular haemoglobin; MCHC, mean corpuscular haemoglobin concentration; RDW, red cell distribution width; Iron, serum iron; Ferritin, serum ferritin; Albumin, serum albumin; Protein, total serum protein; Globulin, serum globulins; Folic acid, serum folate; VitB12, vitamin B12; VitD, vitamin D; Calcium, total serum calcium; Calcium ion, ionized calcium; Zn, zinc; Glu, fasting glucose; HbA1c, glycated haemoglobin; Total cholesterol, total serum cholesterol; TG, triglycerides; HDL, high density lipoprotein cholesterol; Uric acid, serum uric acid; AST, aspartate aminotransferase; ALT, alanine aminotransferase; GGT, gamma glutamyl transferase; CRP, C reactive protein; Creatinine, serum creatinine; TSH, thyroid stimulating hormone; T4, free thyroxine

Participants were stratified into predefined postoperative intervals comprising a preoperative baseline (0 days) and postoperative assessments at 40, 120, 180, and 365 days. Age did not differ significantly across groups (*p* = 0.6230), ranging from 37.8 to 40.8 years, with an overall weighted mean of 38 years. Baseline body weight also exhibited no significant variation among groups, with a mean value of 104.32 kg (*p* = 0.2830). Following surgery, participants demonstrated a progressive and highly significant reduction in body weight across postoperative intervals (*p* < 0.0001). Mean percentage weight loss reached 16.55% at 40 days, 22.94% at 120 days, 25.69% at 180 days, and 33.40% at 365 days, indicating sustained and clinically meaningful postoperative weight reduction throughout the first postoperative year (Fig. 2).

As part of routine postoperative care, some participants received targeted supplementation in addition to standard multivitamin use, including intravenous ferric carboxymaltose, vitamin D, and vitamin B12. These interventions were prescribed in response to clinically relevant laboratory abnormalities during postoperative follow-up and were initiated only after the respective biochemical assessments were performed. During cohort screening, 70 women who received targeted active supplementation capable of directly influencing the biochemical parameters under investigation were identified among the 410 medical records initially assessed for eligibility. To minimize potential confounding effects on the biochemical trajectories evaluated in this study, these patients were excluded from the analytical cohort. Consequently, none of the biochemical measurements included in the analyses were obtained during exposure to active supplementation.

### Haematological Markers

Haematological indices exhibited modest but statistically significant alterations across postoperative intervals. Hemoglobin concentrations did not differ significantly among groups (*p* = 0.0910), remaining within ranges not compatible with overt anaemia throughout the first postoperative year. In contrast, red blood cell count declined progressively and significantly across intervals (*p* < 0.0001), accompanied by a proportional reduction in haematocrit (*p* < 0.0001), although values remained above thresholds typically used to define anaemia. Erythrocyte indices demonstrated discrete but consistent shifts over time. Mean corpuscular volume increased significantly across postoperative intervals (*p* < 0.0001), whereas mean corpuscular hemoglobin (*p* = 0.0160) and mean corpuscular hemoglobin concentration (*p* = 0.0150) showed statistically significant but quantitatively modest variation. Red cell distribution width increased significantly (*p* = 0.0002), with higher values observed predominantly at intermediate postoperative intervals. Similarly, RDW to haemoglobin (*p* = 0.0004) and RDW to haematocrit ratios (*p* = 0.0001) differed significantly across groups, reflecting increased heterogeneity in erythrocyte size during the postoperative period (Fig. 2). Despite these statistically significant changes, the overall haematological profile did not meet criteria for clinically overt haematologic disorders during the first postoperative year, indicating that observed alterations represent subclinical shifts in erythroid parameters rather than established anaemia.

### Iron Status

Markers of iron metabolism demonstrated pronounced and statistically significant alterations across postoperative intervals. Serum iron concentrations increased significantly over time (*p* < 0.0001), with higher values observed in postoperative groups compared with the preoperative baseline. In contrast, ferritin levels declined sharply and progressively across intervals (*p* < 0.0001), with marked reductions already evident at early postoperative assessments and persisting throughout the first postoperative year (Fig. 2). Because of these divergent trajectories, the iron to ferritin ratio increased significantly across groups (*p* < 0.0001), indicating a widening dissociation between circulating iron availability and iron storage status over time. This pattern was further reflected by a significant reduction in the ferritin to C-reactive protein ratio across postoperative intervals (*p* < 0.0001), consistent with concomitant changes in iron stores and systemic inflammatory markers during follow-up. Despite the progressive depletion of ferritin, haematological parameters did not meet criteria for overt iron deficiency anaemia during the study period, indicating that the observed alterations in iron status occurred primarily at the level of iron reserves rather than circulating hemoglobin.

### Protein Markers

Protein related biochemical markers exhibited consistent and statistically significant alterations across postoperative intervals. Serum albumin concentrations declined modestly but significantly over time (*p* = 0.0018), remaining within physiological reference ranges throughout the first postoperative year. Total serum protein levels demonstrated a more pronounced and progressive reduction across groups (*p* < 0.0001), indicating a systematic postoperative shift in circulating protein content. Globulin concentrations also declined significantly across postoperative intervals (*p* < 0.0001), parallelling changes observed in total protein levels (Fig. 2). As a result of these proportional alterations among serum protein fractions, the albumin to globulin ratio differed significantly across groups (*p* < 0.0001), reflecting a redistribution of protein components during the postoperative period. Despite the statistical significance of these changes, no abrupt reductions or patterns consistent with overt protein energy malnutrition were observed during follow-up, indicating that the detected alterations represent gradual postoperative shifts in protein related biochemical markers rather than acute deficiency states.

### Vitamins and Minerals

Markers of vitamin and mineral status demonstrated marked and statistically significant alterations across postoperative intervals. Folate concentrations declined sharply and significantly over time (*p* < 0.0001), with substantial reductions evident at early postoperative assessments and persistently lower levels maintained throughout the first postoperative year. Vitamin B12 levels also varied significantly across groups (*p* < 0.0001), exhibiting a progressive decline across postoperative intervals (Fig. 2). Because of these differential trajectories, the vitamin B12 to folate ratio differed significantly among groups (*p* < 0.0001), reflecting disproportionate changes between these two components of one-carbon metabolism during the postoperative period. In contrast, vitamin D concentrations did not differ significantly across postoperative intervals (*p* = 0.2805), remaining relatively stable throughout follow-up.

Mineral related markers exhibited more modest but statistically significant changes. Total calcium concentrations differed across groups (*p* = 0.0028), with mild postoperative variation that did not reach clinically critical levels. Ionized calcium also varied significantly across intervals (*p* = 0.0070), with slightly higher values observed at later postoperative assessments. The calcium to albumin ratio differed markedly across groups (*p* < 0.0001), reflecting concomitant changes in calcium and protein related markers. Zinc concentrations declined sharply and significantly across postoperative intervals (*p* < 0.0001), with reductions evident early after surgery and persisting throughout the follow-up period. No spontaneous recovery of zinc levels was observed during the first postoperative year (Fig. 2).

### Metabolic Markers

Metabolic parameters demonstrated pronounced and statistically significant improvement across postoperative intervals. Fasting glucose concentrations declined markedly following surgery (*p* < 0.0001), with lower values observed consistently across postoperative groups compared with the preoperative baseline. In contrast, glycated hemoglobin did not differ significantly among groups (*p* = 0.1530), remaining relatively stable throughout the first postoperative year. The absence of significant differences in glycated haemoglobin may be related to its longer biological integration period, reflecting average glycaemic exposure over approximately two to three months. Consequently, HbA1c may be less sensitive to detecting early postoperative metabolic improvements than fasting glucose during periods of rapid physiological adaptation. Lipid profile parameters exhibited substantial postoperative changes. Total cholesterol levels declined significantly across intervals (*p* < 0.0001), accompanied by a marked reduction in triglyceride concentrations (*p* < 0.0001). High density lipoprotein cholesterol differed significantly among groups (*p* = 0.0010), with higher values observed at later postoperative assessments. Consequently, the triglyceride to HDL cholesterol ratio decreased sharply across postoperative intervals (*p* < 0.0001), indicating a consistent shift in lipid related metabolic markers following surgery. Serum uric acid concentrations also declined significantly over time (*p* < 0.0001), with lower values observed across postoperative intervals relative to baseline. These changes occurred in parallel with the observed alterations in glycaemic and lipid markers and were maintained throughout the first postoperative year (Fig. 2).

### Hepatic Function and Inflammation

Markers of hepatic function demonstrated consistent and statistically significant improvement across postoperative intervals. Serum aspartate aminotransferase levels declined markedly following surgery (*p* < 0.0001), accompanied by parallel reductions in alanine aminotransferase (*p* < 0.0001) and gamma glutamyl transferase concentrations (*p* < 0.0001). These reductions were evident early after surgery and were maintained throughout the first postoperative year. Hepatic enzyme ratios also differed significantly among groups. The aspartate aminotransferase to alanine aminotransferase ratio varied across postoperative intervals (*p* < 0.0001), as did the gamma glutamyl transferase to alanine aminotransferase ratio (*p* < 0.0001), reflecting proportional changes among hepatic enzymes during follow-up.

Systemic inflammatory status exhibited a pronounced postoperative shift. C-reactive protein concentrations declined sharply and significantly across postoperative intervals (*p* < 0.0001), with lower values observed at intermediate and late assessments compared with the preoperative baseline. In parallel, the ferritin to C-reactive protein ratio differed significantly across groups (*p* < 0.0001), capturing concurrent changes in inflammatory activity and iron related markers during the postoperative period (Fig. 2).

### Renal and Endocrine Function

Markers of renal and endocrine function exhibited statistically significant but quantitatively modest alterations across postoperative intervals. Serum creatinine concentrations declined significantly over time (*p* < 0.0001), with lower values observed across postoperative groups compared with the preoperative baseline. These changes remained within reference ranges throughout follow-up. Thyroid related markers demonstrated differential postoperative behaviour. Thyroid stimulating hormone levels declined significantly across postoperative intervals (*p* = 0.0003), whereas free thyroxine concentrations did not differ significantly among groups (*p* = 0.3002). Therefore, the thyroid stimulating hormone to free thyroxine ratio differed markedly across postoperative intervals (*p* < 0.0001), reflecting changes in thyrotrophic activity during the postoperative period. No patterns indicative of clinically overt renal or thyroid dysfunction were observed during the first postoperative year, indicating that the detected alterations represent modest postoperative shifts in renal and endocrine related biochemical markers (Fig. 2).

### Exploratory Multivariate Pattern Recognition

**Fig. 3:**
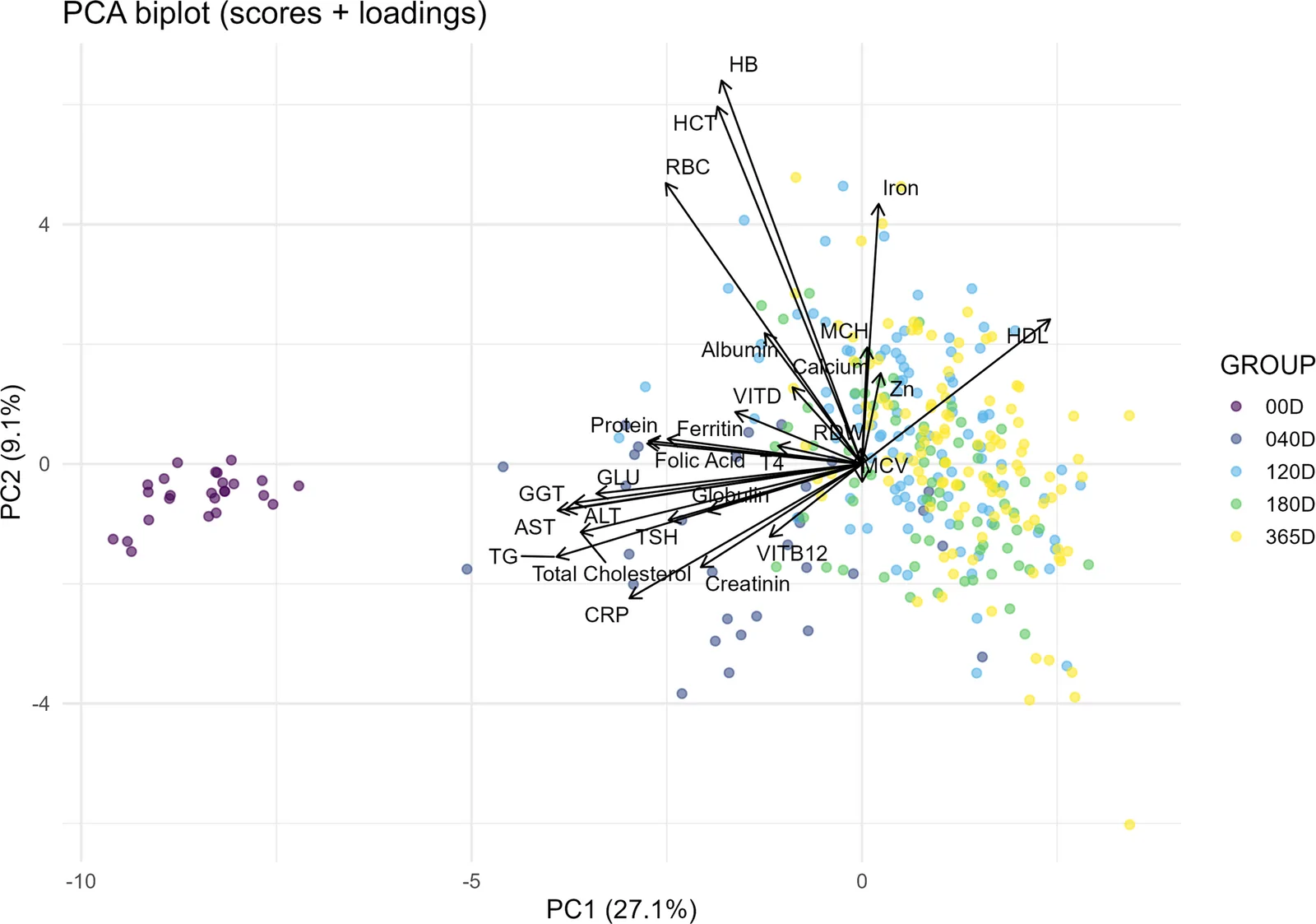
PCA biplot (scores and loadings) for biochemical markers across postoperative intervals (*n* = 340). Principal component analysis was performed on standardized variables (prcomp on z-scores). PC1 and PC2 explain 27.1% and 9.1% of the total variance, respectively. Colored points represent individual samples grouped by postoperative interval: 0 days (purple), 40 days (navy), 120 days (light blue), 180 days (green), 365 days (yellow). Solid arrows denote variable loadings; arrow direction indicates the sign of the loading and arrow length the relative contribution to the component. Top contributors to PC1 (metabolic–hepatic axis): TG (≈ 9.9%), GGT (≈ 9.9%), AST (≈ 9.4%), ALT (≈ 8.8%) and total cholesterol (≈ 8.4%). Top contributors to PC2 (hematologic axis): HB (≈ 26.6%), HCT (≈ 23.2%), RBC (≈ 14.3%) and serum iron (≈ 12.3%)

Principal component analysis (Fig. 3) was applied to the subset of biochemical markers that exhibited significant variation across postoperative intervals. Sampling adequacy was confirmed by Bartlett’s test of sphericity (*p* < 0.0001), indicating sufficient intercorrelation among variables for dimensional reduction. The Kaiser–Meyer–Olkin measure of sampling adequacy demonstrated acceptable suitability for multivariate decomposition (global KMO = 0.76). The first principal component (PC1) presented an eigenvalue of 8.14 and accounted for 27.1% of the total variance. The second principal component (PC2) exhibited an eigenvalue of 2.73 and explained an additional 9.1% of the variance. Subsequent components showed progressively lower eigenvalues (PC3 = 2.22, PC4 = 1.62, PC5 = 1.54), each contributing between 7.4% and 3.4% of the variance. The cumulative variance explained by the first two components reached 36.2%, consistent with the multidimensional structure expected for heterogeneous physiological data.

PC1 was primarily characterized by metabolic and hepatic markers, with the highest absolute loadings observed for triglycerides (− 0.71), gamma glutamyl transferase (− 0.68), aspartate aminotransferase (− 0.64), alanine aminotransferase (− 0.62), and glucose (− 0.59). PC2 was predominantly defined by haematological variables, with strong positive loadings for hemoglobin (0.74), haematocrit (0.71), red blood cell count (0.68), and mean corpuscular volume (0.63), indicating an independent erythroid dimension within the multivariate structure.

In the PCA biplot, samples from the preoperative baseline group (G0D) clustered compactly on the negative side of PC1, displaying limited dispersion. Postoperative samples were progressively displaced along the PC1 axis across follow-up intervals, forming broader but coherent groupings at 40, 120, and 365 days. Elliptical confidence regions demonstrated partial overlap among postoperative groups but complete separation between baseline and all postoperative timepoints. PC2 contributed secondary differentiation without substantial separation among postoperative intervals. Overall, PCA identified two dominant and physiologically interpretable axes of variation, corresponding to a metabolic–hepatic dimension captured by PC1 and a haematological dimension represented by PC2. Although the variance explained by the first two components was moderate, the ordination revealed a consistent multivariate structure characterized by clear baseline separation and organized postoperative displacement across biochemical domains.

### Multivariate physiological remodelling across postoperative intervals

**Fig. 4:**
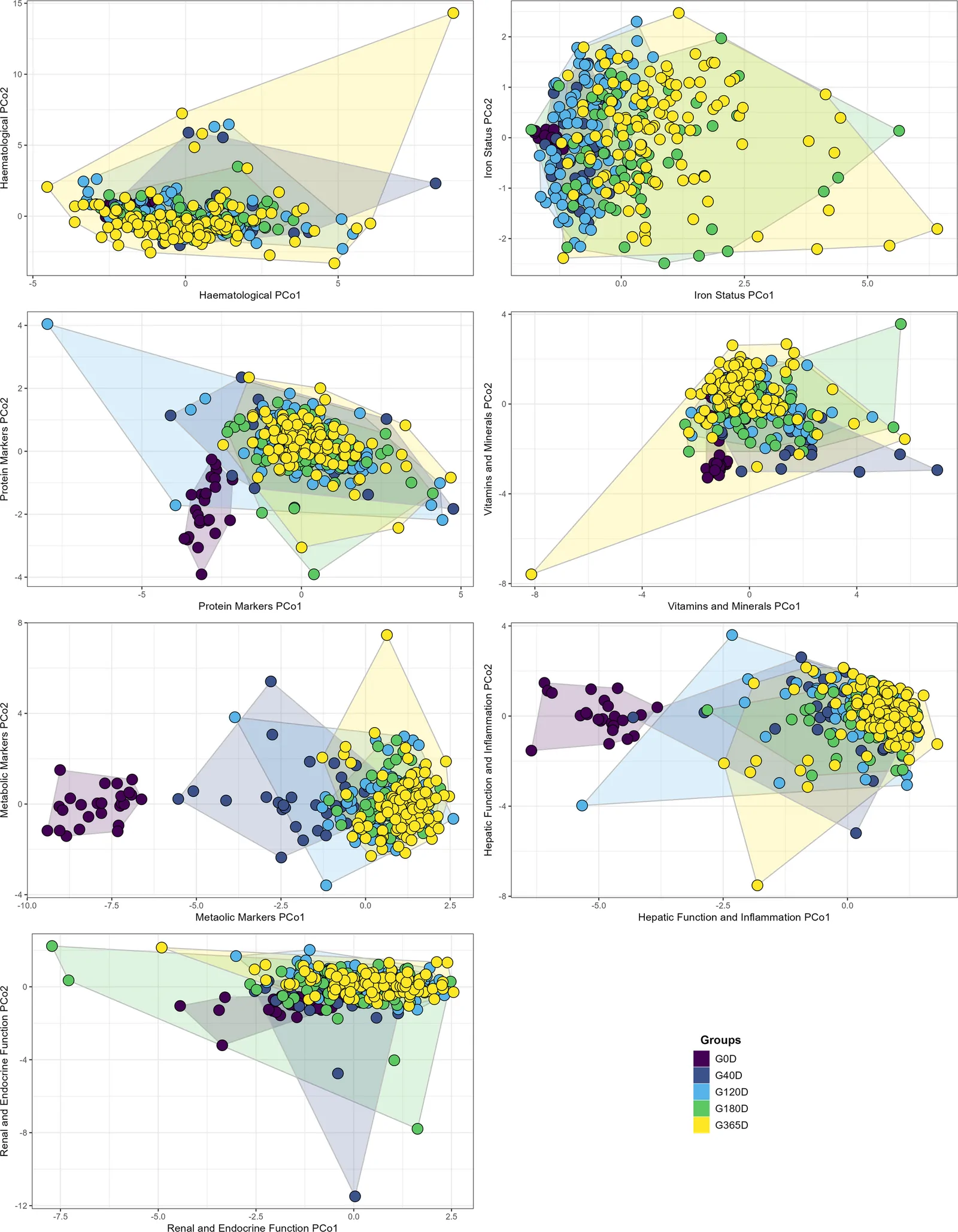
Multivariate ordination of physiological systems across postoperative intervals: Principal coordinates analysis (PCoA) of standardized biochemical markers grouped into physiological panels: haematological markers, iron status, protein markers, vitamins and minerals, metabolic markers, hepatic function and inflammation, and renal and endocrine function (*n* = 340). For each panel, Euclidean distance matrices were computed from z-scored variables, and group-level differences were evaluated using PERMANOVA. Colored points represent individual samples according to postoperative interval: preoperative baseline (G0D, purple), 40 days (G40D, navy), 120 days (G120D, light blue), 180 days (G180D, green), and 365 days (G365D, yellow)

Multivariate analyses revealed pronounced and system-wide physiological remodelling across postoperative intervals (Fig. 4). Distance-based ordination using principal coordinates analysis (PCoA) demonstrated consistent separation between the preoperative baseline group (G0D) and all postoperative groups across all physiological panels. These visual patterns were formally supported by permutational multivariate analysis of variance (PERMANOVA), which identified significant multivariate effects of postoperative group membership for haematological markers (pseudo-F = 4.29, R² = 0.0487, *p* < 0.0001), iron status (pseudo-F = 26.47, R² = 0.2402, *p* < 0.0001), protein markers (pseudo-F = 27.73, R² = 0.2488, *p* < 0.0001), vitamins and minerals (pseudo-F = 32.72, R² = 0.2810, *p* < 0.0001), metabolic markers (pseudo-F = 86.39, R² = 0.5078, *p* < 0.0001), hepatic function and inflammation (pseudo-F = 57.25, R² = 0.4060, *p* < 0.0001), and renal and endocrine function (pseudo-F = 17.41, R² = 0.1721, *p* < 0.0001). Collectively, these results indicate that postoperative status accounted for a significant proportion of multivariate variance across all physiological systems analysed, with effect sizes varying substantially by domain.

Pairwise post hoc PERMANOVA analyses demonstrated that this global separation was driven primarily by differences between the baseline group (G0D) and each postoperative interval. In the haematological panel, all baseline versus postoperative comparisons were significant after Bonferroni correction (G0D vs. G40D: pseudo-F = 10.82, R² = 0.1670, p_adj < 0.001; G0D vs. G120D: pseudo-F = 12.57, R² = 0.0941, p_adj < 0.001; G0D vs. G180D: pseudo-F = 25.98, R² = 0.2148, p_adj < 0.001; G0D vs. G365D: pseudo-F = 11.01, R² = 0.0744, p_adj < 0.001), whereas no significant differences were observed among postoperative groups themselves, indicating that haematological reorganization occurred early relative to surgery and remained stable thereafter.

For iron status, baseline comparisons with all postoperative intervals were also highly significant (G0D vs. G40D: pseudo-F = 16.26, R² = 0.2315, p_adj < 0.001; G0D vs. G120D: pseudo-F = 14.79, R² = 0.1089, p_adj < 0.001; G0D vs. G180D: pseudo-F = 37.41, R² = 0.2826, p_adj < 0.001; G0D vs. G365D: pseudo-F = 43.53, R² = 0.2411, p_adj < 0.001). In contrast to the haematological panel, selected comparisons among postoperative groups remained significant, particularly those involving the early postoperative period, indicating a more dynamic and temporally structured remodelling of iron metabolism following surgery.

Protein markers exhibited a similar baseline-driven pattern, with strong and significant separation between G0D and all postoperative groups (pseudo-F range = 61.45–148.54; R² range = 0.404–0.522; all p_adj < 0.001), while comparisons among postoperative intervals were not statistically significant after correction. This pattern indicates a substantial shift in protein-related multivariate profiles following surgery, followed by relative stabilization across later postoperative timepoints.

In the vitamins and minerals panel, baseline samples differed significantly from all postoperative groups (pseudo-F range = 29.18–85.81; R² range = 0.223–0.415; all p_adj < 0.001), and several comparisons among postoperative intervals also remained significant after adjustment. These findings indicate that micronutrient profiles underwent both an immediate postoperative shift and continued restructuring across later timepoints.

The metabolic panel showed the strongest baseline-centered divergence of all systems analysed. Comparisons between G0D and each postoperative group yielded very large effect sizes (pseudo-F range = 82.59–276.05; R² range = 0.605–0.743; all p_adj < 0.001), indicating profound metabolic reorganization following surgery. Additionally, several postoperative comparisons remained significant, particularly those involving the earliest postoperative interval, supporting the presence of a graded metabolic trajectory.

Similarly, hepatic function and inflammatory markers displayed marked separation between baseline and postoperative groups (pseudo-F range = 108.53–290.76; R² range = 0.581–0.754; all p_adj < 0.001), while most postoperative comparisons were not significant after correction. This pattern indicates that hepatic and inflammatory remodelling was largely established early and maintained throughout follow-up.

Finally, renal and endocrine markers also differed significantly between G0D and all postoperative intervals (pseudo-F range = 64.01–87.52; R² range = 0.332–0.420; all p_adj < 0.001). Comparisons among postoperative groups were fewer and generally of smaller magnitude, indicating moderate but persistent renal and endocrine adaptation following surgery.

### Integrated Trajectory of Nutritional and Metabolic Adaptation

**Fig. 5:**
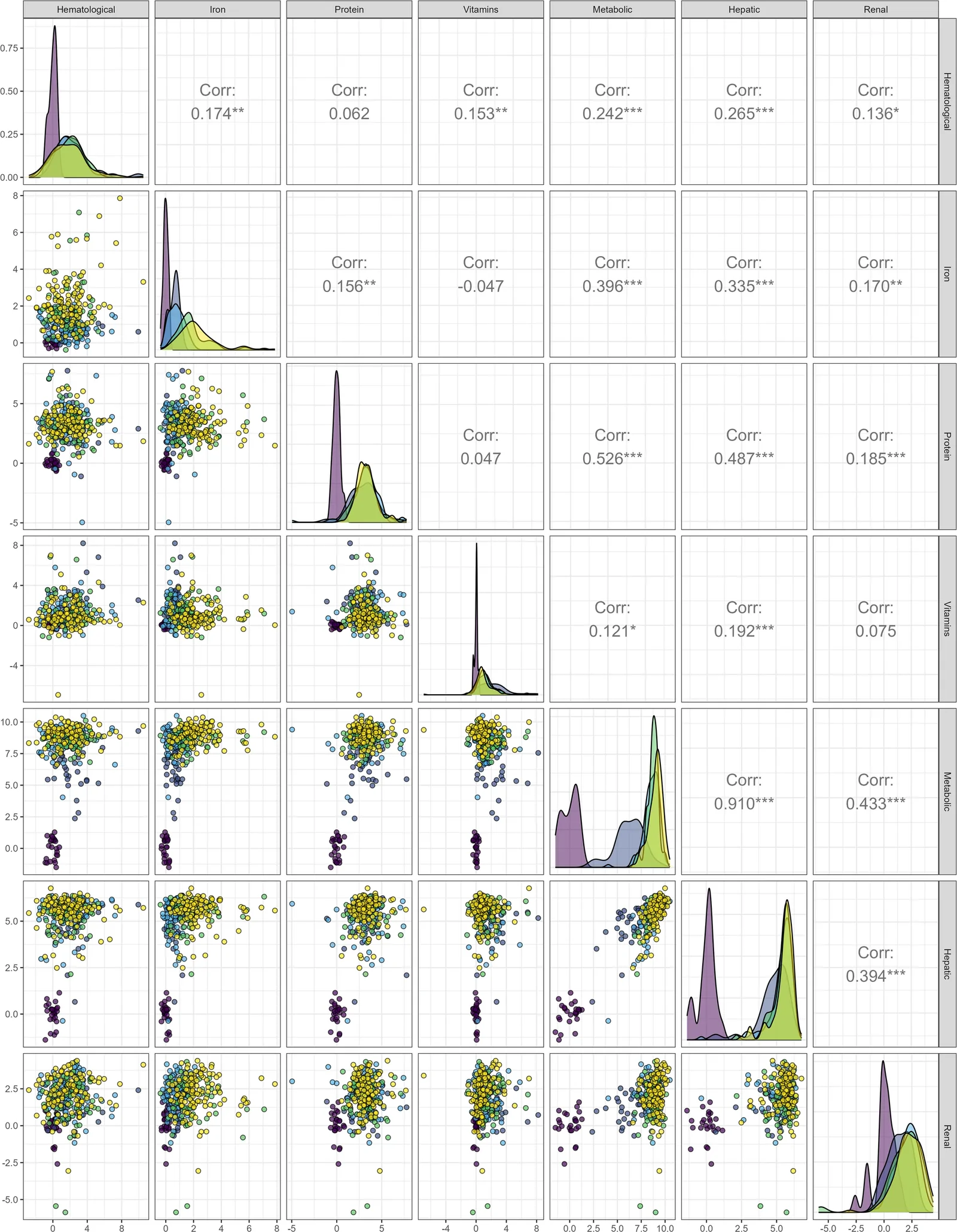
Multivariate scatterplot matrix (SPLOM) depicting cross-system relationships among physiological panels across postoperative intervals: Pairwise associations among the multivariate scores derived from each physiological panel (haematological, iron, protein, vitamins and minerals, metabolic, hepatic, and renal–endocrine) are displayed as a scatterplot matrix. Diagonal panels show kernel density distributions for each physiological domain, stratified by postoperative group: 0 days (G0D, purple), 40 days (G40D, navy), 120 days (G120D, light blue), 180 days (G180D, green), and 365 days (G365D, yellow). Off-diagonal panels present pairwise scatterplots of individual samples, illustrating coordinated shifts across physiological systems following surgery. Upper panel’s report Pearson correlation coefficients (r) between panel scores, with statistical significance denoted as *p* < 0.05 (*), *p* < 0.01 (**), and *p* < 0.001 (***)

Across the 12 month follow-up, scatterplot matrix analysis was used to examine cross system relationships among multivariate scores derived from each physiological panel (Fig. 5). At the preoperative baseline, samples exhibited compact and overlapping distributions across panels, with strong positive correlations observed among metabolic, hepatic, protein, and renal–endocrine domains, indicating a coherent multivariate physiological state prior to surgery.

Following surgery, postoperative samples demonstrated coordinated displacement along a dominant metabolic–hepatic dimension. This pattern was reflected by a strong positive correlation between metabolic and hepatic panel scores (*r* = 0.910, *p* < 0.001), which was consistently observed across all postoperative intervals. In addition to this dominant axis, moderate correlations were detected between metabolic and protein panels (*r* = 0.526, *p* < 0.001) and between metabolic and renal–endocrine panels (*r* = 0.433, *p* < 0.001), indicating secondary coupling between systemic metabolic status and protein related and renal–endocrine domains

Intermediate levels of integration were observed for iron and protein related panels. Iron status exhibited moderate correlations with metabolic (*r* = 0.396, *p* < 0.001) and hepatic domains (*r* = 0.335, *p* < 0.001), accompanied by weaker associations with protein (*r* = 0.156, *p* < 0.01) and renal–endocrine panels (*r* = 0.170, *p* < 0.01). Protein panel scores were moderately correlated with metabolic (*r* = 0.526, *p* < 0.001) and hepatic panels (*r* = 0.487, *p* < 0.001), while showing weak or non-significant associations with haematological (*r* = 0.062) and vitamin–mineral panels (*r* = 0.047). These patterns reflect asymmetric integration, with protein and iron domains aligning more closely with metabolic and hepatic systems than with micronutrient or erythroid domains.

In contrast, micronutrient and haematological panels demonstrated consistently weak coupling with most other physiological systems. Vitamins and minerals exhibited only modest associations with metabolic scores (*r* = 0.121, *p* < 0.05) and no significant correlation with protein panels, while associations with hepatic (*r* = 0.192, *p* < 0.001) and renal–endocrine domains (*r* = 0.075) were similarly limited. Haematological panel scores showed small correlation coefficients across all domains, including iron (*r* = 0.174, *p* < 0.01), metabolic (*r* = 0.265, *p* < 0.001), and hepatic panels (*r* = 0.265, *p* < 0.001), indicating limited shared multivariate variance despite statistical significance in selected comparisons.

Across postoperative intervals, samples showed increasing overlap within nutritional and haematological panels, while maintaining clear separation from the preoperative baseline in metabolic and hepatic dimensions. This pattern was visually evident as broader postoperative dispersion for vitamins, minerals, and protein panels relative to compact baseline distributions, contrasted with tightly aligned postoperative trajectories in metabolic and hepatic panels.

At 365 days, postoperative samples occupied a stable region of ordination space in metabolic and hepatic panels, whereas nutritional and haematological panels remained more widely dispersed with weaker cross panel correlations. Together, these results describe a hierarchical multivariate structure characterized by a tightly integrated metabolic–hepatic core, moderately associated protein, iron, and renal–endocrine domains, and comparatively weaker integration of micronutrient and haematological systems across the first postoperative year.

## Discussion

The present study identifies a robust and clinically relevant dual trajectory during the first postoperative year after sleeve gastrectomy in women. On one hand, participants exhibited substantial and progressive improvement in metabolic, hepatic, and inflammatory parameters. On the other, a sustained deterioration in several micronutrient and protein related markers was observed, particularly folate, vitamin B12, ferritin, and zinc. Together, these findings indicate that metabolic recovery and nutritional status follow partially dissociated trajectories after surgery, evolving with different temporal patterns and degrees of integration. Consequently, metabolic improvement should not be interpreted as a reliable surrogate for nutritional adequacy at the population level, underscoring an important dimension of postoperative care that may be underestimated in routine clinical practice.

Consistent with previous literature, weight loss was pronounced and aligned with expected outcomes following sleeve gastrectomy [12]. Although the present study did not directly assess hormonal mediators, existing evidence suggests that metabolic improvements after sleeve gastrectomy are strongly influenced by neuroendocrine adaptations, including reductions in ghrelin secretion and enhanced incretin signalling through glucagon-like peptide 1 and peptide YY [2, 8, 38, 51]. These adaptations are associated with early appetite suppression, improved glucose homeostasis, and favourable modulation of lipid metabolism. In this context, the rapid decline in fasting glucose observed as early as 40 days postoperatively is consistent with incretin mediated enhancement of pancreatic β cell responsiveness and peripheral insulin sensitivity described in experimental and clinical studies [32, 37]. Continued weight loss over subsequent months has been associated with further reductions in insulin resistance, which may contribute to the sustained improvements observed in glycaemic markers and to the concomitant decline in serum uric acid, a parameter linked to oxidative stress and purine metabolism [5].

Postoperative remodelling of lipid metabolism was also evident, with reductions in total cholesterol and triglycerides accompanied by increases in high density lipoprotein cholesterol. In the present study, the term metabolic–hepatic remodelling refers to the coordinated improvement observed across glycaemic, lipid, hepatic, and inflammatory biomarkers following surgery, reflecting integrated physiological adaptations associated with weight loss, improved insulin sensitivity, reduced hepatic fat accumulation, and attenuation of chronic systemic inflammation [14, 24, 28, 61]. These changes are characteristic of metabolic adaptation following bariatric surgery and have been linked to improved insulin signalling and reduced hepatic lipid accumulation [31, 36, 61]. The marked decline in hepatic enzymes observed in the present cohort is consistent with attenuation of obesity associated hepatic dysfunction reported in previous studies and may reflect reduced lipid influx into hepatocytes, diminished inflammatory burden, and improved mitochondrial efficiency [14, 24, 28, 32].

The coordinated reduction in AST, ALT, and GGT further supports the concept of integrated metabolic–hepatic remodelling following sleeve gastrectomy, suggesting progressive improvement in hepatocellular function accompanying systemic metabolic recovery [7, 15, 44]. In parallel, the reduction in C-reactive protein supports a decrease in chronic low grade systemic inflammation, a hallmark of severe obesity, often attributed to reductions in visceral adiposity and inflammatory cytokine production [14, 24, 31]. The observed decline in thyroid stimulating hormone is also consistent with literature suggesting partial normalization of hypothalamic pituitary thyroid axis activity following weight loss, given that obesity related hyperleptinemia can elevate TSH independently of intrinsic thyroid disease [3, 27, 41, 56]. Small decreases in serum creatinine, as previously reported, are more plausibly explained by reductions in lean body mass than by impaired renal function [1, 10, 23, 49].

In contrast to the metabolic trajectory, micronutrient status deteriorated early after surgery and remained compromised throughout the 12-month follow-up. This pattern aligns with previous reports identifying the postoperative period as a phase of heightened nutritional vulnerability due to reduced gastric acidity, diminished intrinsic factor secretion, restricted intake of nutrient dense foods, and altered gastrointestinal physiology affecting absorption efficiency [22, 34, 55]. The early and persistent reduction in folate concentrations, evident by 40 days and sustained throughout follow-up, is consistent with impaired jejunal absorption and insufficient dietary intake, processes that may impact one-carbon metabolism, erythropoiesis, and homocysteine regulation [9, 25, 35, 59]. Vitamin B12 levels declined progressively, in agreement with reductions in intrinsic factor availability and compromised ileal absorption described after sleeve gastrectomy [21, 35].

Iron metabolism displayed a nuanced and clinically informative dissociation. While serum iron concentrations increased over time, ferritin levels declined progressively, indicating depletion of iron stores despite preserved or elevated circulating iron. This pattern is biologically plausible, as ferritin reflects intracellular iron reserves, whereas serum iron is more sensitive to short term absorption and mobilization dynamics. Reduced gastric acidity, decreased intake of heme rich foods, and altered absorption in the duodenum and proximal jejunum likely contribute to declining ferritin stores after surgery [2, 11, 35, 45, 48, 59]. Although overt iron deficiency anaemia was not observed during the first postoperative year, the downward trajectory of ferritin suggests increased vulnerability over longer follow-up periods, as reported in longitudinal studies [52, 53].

Protein related markers exhibited modest but consistent reductions, particularly in the early postoperative period, likely reflecting reduced intake during rapid weight loss phases. Although albumin and globulin concentrations stabilized over time and remained within reference ranges, persistent reductions may have implications for immune competence and tissue repair [11]. Interpretation of circulating micronutrient concentrations should also consider the potential influence of systemic inflammatory status and serum protein concentrations. Previous studies have demonstrated that inflammatory activity, particularly when accompanied by elevated C-reactive protein levels, as well as alterations in albumin concentrations, may affect circulating concentrations of selected vitamins and trace elements independently of true nutritional status. Although the progressive reduction in C-reactive protein observed in the present cohort suggests attenuation of systemic inflammation throughout follow-up, these factors should be considered when interpreting postoperative micronutrient trajectories in bariatric populations [13, 19, 39]. The sustained decline in zinc concentrations is noteworthy given its role as a cofactor in numerous enzymatic processes related to immune function, antioxidant defence, and wound healing [48]. Calcium and vitamin D levels remained near the lower limits of normal, a finding of clinical relevance considering the high prevalence of baseline hypovitaminosis D in obesity and the reduced absorption of fat-soluble vitamins after bariatric surgery. Over longer timeframes, such trajectories may contribute to alterations in bone mineral metabolism and fracture risk [4, 54, 62].

Taken together, the observed patterns indicate a clear physiological dissociation between rapid, coordinated metabolic remodelling and slower, more heterogeneous nutritional deterioration. While metabolic systems appear to reorganize in a tightly coupled manner, nutritional and haematological domains exhibit weaker integration and limited spontaneous recovery. These findings support the view that postoperative management should extend beyond metabolic monitoring to include sustained nutritional surveillance, individualized supplementation strategies, and early intervention to mitigate long term complications.

Additionally, the retrospective real-world design resulted in heterogeneous follow-up completeness across postoperative intervals. Patients contributing laboratory data to later follow-up stages may represent a clinically distinct subgroup enriched for greater adherence to monitoring, persistent postoperative care, suspected nutritional deficiencies, or clinical complications. Therefore, follow-up related selection bias should be considered when interpreting long-term biochemical trajectories.

Several limitations should be acknowledged. The cohort included only women, limiting generalizability to other populations. The 12 month follow-up, while clinically informative, may underestimate the magnitude of nutritional deficiencies that often intensify beyond the first postoperative year. Adherence to supplementation protocols was not quantified, and dietary intake and body composition were not assessed, precluding direct linkage between biochemical trajectories and behavioural or physiological determinants. Future studies should incorporate longer follow-up, objective assessment of adherence, dietary quality, hormonal profiles, and comparative analyses across bariatric techniques. Additionally, information regarding dietary adherence, oral contraceptive use, gastrointestinal motility disorders, laxative use, small intestinal bacterial overgrowth, preoperative rehabilitation protocols, surgical standardization, leptin concentrations, cortisol levels, and basal metabolic rate was not consistently available within the retrospective clinical records and therefore could not be systematically evaluated.

## Conclusion

In conclusion, sleeve gastrectomy in women is associated with robust and sustained metabolic improvement during the first postoperative year, accompanied by early, progressive, and persistent deterioration of key micronutrient and protein-related markers. These findings demonstrate that metabolic recovery and nutritional adequacy represent partially dissociated physiological processes following surgery, evolving with distinct temporal dynamics. Importantly, improvements in metabolic and inflammatory parameters do not reliably reflect underlying nutritional status, particularly during the early postoperative period. This dissociation has direct translational implications for postoperative care, indicating that follow-up strategies centred primarily on metabolic outcomes may be insufficient to detect clinically relevant nutritional vulnerability during postoperative follow-up. Our results support the need for high-frequency, laboratory-based nutritional surveillance and individualized supplementation protocols beginning early after surgery, with the goal of preventing subclinical deficiencies before overt clinical manifestations arise. These findings further reinforce the importance of integrated multidisciplinary postoperative follow-up, including nutritional, metabolic, and psychological support aimed at early detection and management of postoperative nutritional and systemic complications. Integrating comprehensive biochemical monitoring into routine postoperative care may contribute to improved long-term health outcomes and refinement of current standards of care following sleeve gastrectomy.

## Data Availability

The data supporting this study are available at: Zazula, Matheus; Bonfim de Oliveira, Alana; Piovezan Pereira, Gabrielly Eduarda ; Fonteles de Moura, Thamira ; dos Santos, Wilson Paulo; Maciel, Mônica; Donatti, Lucélia; Fernandes, Luiz Claudio; Naliwaiko, Katya; Manzano Watanabe, Mariana Inocêncio (2026), “Obesity Sleeve Gastrectomy”, Mendeley Data, V2, doi: 10.17632/j6d3vkrtn3.2.
